# FIN-PRINT a fully-automated multi-stage deep-learning-based framework for the individual recognition of killer whales

**DOI:** 10.1038/s41598-021-02506-6

**Published:** 2021-12-06

**Authors:** Christian Bergler, Alexander Gebhard, Jared R. Towers, Leonid Butyrev, Gary J. Sutton, Tasli J. H. Shaw, Andreas Maier, Elmar Nöth

**Affiliations:** 1 Department of Computer Science - Pattern Recognition Lab, Friedrich-Alexander-University Erlangen-Nuremberg, Martensstr. 3, 91058 Erlangen, Germany; 2 Bay Cetology, 257 Fir street, Alert Bay, BC V0N 1A0 Canada; 3 Pacific Biological Station, Fisheries and Oceans Canada, 3190 Hammond Bay Road, Nanaimo, BC V9T 6N7 Canada

**Keywords:** Marine biology, Ecology, Machine learning

## Abstract

Biometric identification techniques such as photo-identification require an array of unique natural markings to identify individuals. From 1975 to present, Bigg’s killer whales have been photo-identified along the west coast of North America, resulting in one of the largest and longest-running cetacean photo-identification datasets. However, data maintenance and analysis are extremely time and resource consuming. This study transfers the procedure of killer whale image identification into a fully automated, multi-stage, deep learning framework, entitled FIN-PRINT. It is composed of multiple sequentially ordered sub-components. FIN-PRINT is trained and evaluated on a dataset collected over an 8-year period (2011–2018) in the coastal waters off western North America, including 121,000 human-annotated identification images of Bigg’s killer whales. At first, object detection is performed to identify unique killer whale markings, resulting in 94.4% recall, 94.1% precision, and 93.4% mean-average-precision (mAP). Second, all previously identified natural killer whale markings are extracted. The third step introduces a data enhancement mechanism by filtering between valid and invalid markings from previous processing levels, achieving 92.8% recall, 97.5%, precision, and 95.2% accuracy. The fourth and final step involves multi-class individual recognition. When evaluated on the network test set, it achieved an accuracy of 92.5% with 97.2% top-3 unweighted accuracy (TUA) for the 100 most commonly photo-identified killer whales. Additionally, the method achieved an accuracy of 84.5% and a TUA of 92.9% when applied to the entire 2018 image collection of the 100 most common killer whales. The source code of FIN-PRINT can be adapted to other species and will be publicly available.

## Introduction

Biometric recognition typically relies on the visual differentiation of unique features on specific body parts of individuals. The best-known examples to distinguish identities of humans include analysis of individual fingerprint designs, retina features, and the composition of facial components^1,2^. Individual recognition is also important in the field of wildlife biology, where images of specific body features are systematically used to differentiate between individuals of the same species. For example, repeated photo-identification of pigment patterns and appendage shape on individuals of various species of invertebrate^3^, aquatic^4^ and terrestrial mammals^5,6^, birds^7^, fish^8,9^, reptiles^10,11^, and amphibians^12,13^ can be used to gain insights into the abundance, range, behaviour, ecology, and health of their populations.

The first systematic efforts to photo-identify free-ranging cetaceans began in the early 1970s^4^ and included studies on the population abundance of killer whales off the west coast of Canada^14^. It was found that individuals of this species could be recognized by the unique shapes of their dorsal fins as well as the shapes and pigment patterns on their saddle patches that were visible when the whales came to the surface. Thus, a combination of both attributes (dorsal fin and saddle patch) provides a distinct identification criterion^15^. Over time, several sympatric but genetically and behaviourally distinct populations of killer whales were discovered in the eastern North Pacific using photo-identification^16^. The “west coast transient” population of Bigg’s killer whales is currently among the largest and most commonly photo-identified killer whale populations in this region. Individuals have been systematically, but opportunistically photo-identified, from either or both, the left and right side, each year from 1975 to present resulting in one of the largest and longest-running cetacean photo-identification data archives in existence^15^.

The management and analysis of these photo-identification data currently require manual efforts which include labeling and sorting images, applying identification metadata to each photo^17^, entering resulting information into databases, and the periodic publication of reference material^15^. These tasks are typically best performed only by those who are intimately familiar with the unique physical features and social patterns of individuals in this population, as well as how they are likely to change over time. However, this requires an exceptional level of speciality and amount of time that may be expedited by taking advantage of developing technologies. Computers have assisted efforts to discern identities of individual cetaceans in identification images since the 1980s^18,19^ and over the following decades have been used increasingly to help manage workflow^20^ and automate image analysis processes^21–24^.

Most recently, machine (deep) learning algorithms have been setting new standards for image processing/analysis across various research areas and fields of application^25–33^, due to increasing memory space and performance of central processing units (CPU) and graphics processing units (GPU)^34–37^. Among many other image processing problems handled by deep learning, deep neural networks have recently also been applied to the detection and classification of individual animals of several species including amur tigers (*Panthera tigris altaica*)^38–41^, elephants (*Proboscidea*)^42^, right whales (*Eubalaena*)^43,44^, humpback whales (*Megaptera novaeangliae*)^45–47^, brown bears (*Ursus arctos*)^48^, giraffes (*Giraffa camelopardalis*)^49^, pigs (*Sus scrofa domesticus*)^50^, manta rays (*Mobula birostris*)^51^, common dolphins (*Delphinus delphis*)^52^, chimpanzees (*Pan troglodytes verus*)^53^, red pandas (*Ailurus fulgens*)^54^, giant pandas (*Ailuropoda melanoleuca*)^55^, birds (e.g. sociable weaver (*Philetairus socius*), great tit (*Parus major*), zebra finch (*Taeniopygia guttata*))^56^, gorillas (*Gorilla*)^57^, primates (e.g. rhesus macaque (*Macaca mulatta*))^58^, cattle (*Bos taurus*)^59^, kiangs (*Equus kiang*)^60^, zebras (*Equus quagga*) and nyalas (*Tragelaphus angasii*)^61^, hawksbills (*Eretmochelys imbricata*)^62^, blue whales (*Balaenoptera musculus*)^63^, and common bottlenose dolphins (*Tursiops truncatus*)^64^. Besides deep learning-based detection and identification studies on single animal species, recent research also addresses cross-species recognition^65–70^.

**Figure 1:**
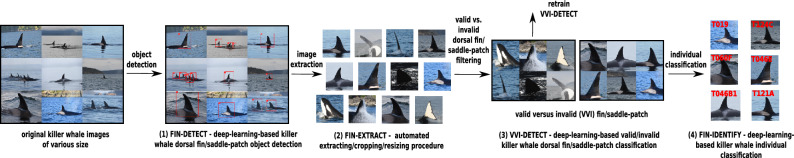
FIN-PRINT workflow including: (1) dorsal fin/saddle patch detection, (2) extraction of the detected killer whale markings, (3) valid versus invalid dorsal fin/saddle patch binary classification, and (4) multi-class killer whale individual identification.

Despite some promising studies in the field of machine (deep) learning, it is difficult to transfer and apply existing approaches to model an end-to-end killer whale individual recognition pipeline, consisting of detection, extraction, enhancement, and classification (see Fig. 1). Several studies perform animal identification across different species^65–70^, rather than recognition of individuals belonging to the same species. Others address only parts of an individual identification pipeline, such as only detection^60^ or classification^59,68,69^. Some approaches present a combination of modern deep learning techniques together with traditional machine learning algorithms^39,42,48–51,57,69^. FIN-PRINT provides a modular, transferable, and state-of-the-art identification pipeline for killer whales, exclusively applying well-established deep learning concepts, to facilitate robust and task-specific feature learning at each stage. In comparison to traditional machine learning methods, all features were learned and derived in a data-driven fashion. Consequently, it was not necessary to perform any feature selection based on heuristic and/or analytical approaches. FIN-PRINT was trained and evaluated on a large, variable and complex dataset of approximately 121,000 human-annotated Bigg’s killer whale identification images. In order to robustly handle the diversity in this dataset, FIN-PRINT integrates an automated, deep learning-based quality inspection, acting as a validation mechanism prior to the final classification. This guarantees that both the original image and the results obtained from upstream steps (e.g. detection), meet the standards for robust individual classification. A number of studies performed Deep Metric Learning along with the triplet loss^71–73^, modifications of it, and/or combinations with other loss functions^38,40,45,46,49,51,52,64^. However, specification of appropriate hard and semi-hard triplets^73^ is extremely challenging, since: (1) killer whale individuals have been recorded from both body sides, resulting in different animal orientations besides potential deviating natural markings^45^, (2) natural identifiers change over time (growth, acquisition of scars, etc.), (3) deviating saddle patch visibility, often obscured to some extent by water and/or other animals, as well as (4) variation of challenging image conditions (e.g. weather, exposure, etc.). Due to the mentioned difficulties, next to sufficiently large individual-specific data volumes, traditional supervised multi-class classification was applied to build an initial pilot system.

The FIN-PRINT pipeline (see Fig. 1) consists of (1) FIN-DETECT, a YOLOv3^74–77^ -based object detection network for recognizing killer whale dorsal fins and associated saddle patches in images with 1 to N individuals, (2) FIN-EXTRACT, an automatic extraction procedure cropping and equally resizing all detected dorsal fin/saddle patch markings within an image, (3) VVI-DETECT, a ResNet34^78^-based convolutional neural network (CNN) performing data enhancement by classifying between previously detected/extracted valid versus invalid (VVI) killer whale identification sub-images (e.g. bad weather conditions, blurred, missing saddle patch, difficult angle, detection errors, etc.), and (4) FIN-IDENTIFY, a ResNet34^78^-based CNN for multi-class killer whale individual classification modeling the 100 most commonly photo-identified killer whales. To the best of the authors’ knowledge, this is the first study transferring the analysis of killer whale image identification^15^ into a fully automated, multi-stage, sequentially ordered, deep-learning-based framework, in order to machine-identify individuals.

## Materials and methods

### Bigg’s killer whale photo-identification dataset

The dataset of this study includes photos of Bigg’s killer whale individuals accumulated over a period of 8 years (2011–2018), from the coastal waters of southeastern Alaska down to central California^15^. None of these animals were directly approached explicitly for this study. All photo-identification data was collected under federally authorized research licenses or from beyond mandated minimum viewing distances.

Supplementary Figure S1 visualizes a series of example images of this dataset. Each image contains one or more individuals. In addition to the identification name of the individual(s), further metadata such as photographer, GPS-coordinates, date, and time are provided. Every identification label is an alphanumeric sequence based on the animals’ ecotype (T—Transient), order of original documentation (e.g. T109), and order of birth (e.g. T109A2—the second offspring of the first offspring of T109)^15^.

**Figure 2:**
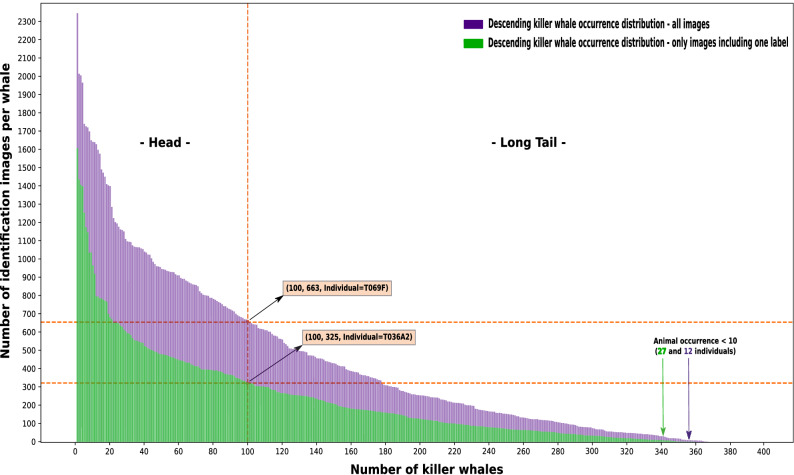
Bigg’s killer whale image long-tailed data distribution (2011–2018), summing up a total of 121,095 identification images, with 86,789 containing single labels, as well as 34,306 photos including multiple labels, resulting in 367 identified individuals (average number of images per individual $\approx$456, standard deviation $\approx$442). The two colored graphs visualize the number of identification images per whale in descending order w.r.t. all images, including single and multiple labels (purple curve) and those only containing a single label (green curve). Furthermore, an exemplary data point is visualized for both curves, presenting the number of identification images in relation to a selected number of whales, here for the top-100, clearly describing the exponential decline. Moreover, the number of animals at which the total amount of identification images is < 10 were marked for both curves. In total, 367 individuals were encountered across 2011–2018. Among them, 128 and 125 were found at least once in each year when considering all images and only those with single labels, respectively.

A parsing procedure was designed to verify, analyze, and prepare the image data, guaranteeing adequate preparation for subsequent machine (deep) learning methods. Results of the entire data parsing procedure are presented in Fig. 2 and Supplementary Table S1. Figure 2 visualizes the number of identified individuals, together with the total amount of occurrences in descending order, considering (1) all images, and (2) only photos including a single label. General statistics with respect to the entire dataset are reported in the caption of Fig. 2. Supplementary Table S1 illustrates the 10 most commonly occurring individuals across all 8 years of data, considering all images including single and multiple labels, compared to photos only containing a single label.

The dataset exhibits a substantial class imbalance, as evidenced by the exponential decline in frequencies per killer whale individual (see Fig. 2). Especially for real-world datasets, such unbalanced data partitioning is a common and well-known phenomenon, also referred to as long-tailed data distribution^79^. Such long-tailed data distributions are divided into two sections^79^: (1) the *Head* region—representing the most commonly identified killer whale individuals, and (2) the *Long-Tail* region—visualizing a significantly larger number of killer whale individuals, however, with considerably less occurrences. For the purpose of this pilot study, the top-100 most commonly occurring killer whale individuals were selected for supervised classification and as boundary between the head and long-tail area (see Fig. 2). The defined boundary of the top-100 killer whales (head region) represents approximately 1/4 (100 out of 367) of the individuals, however, covering about 2/3 (55,305 out of 86,789) of the entire dataset of single-labeled images.

**Figure 3:**
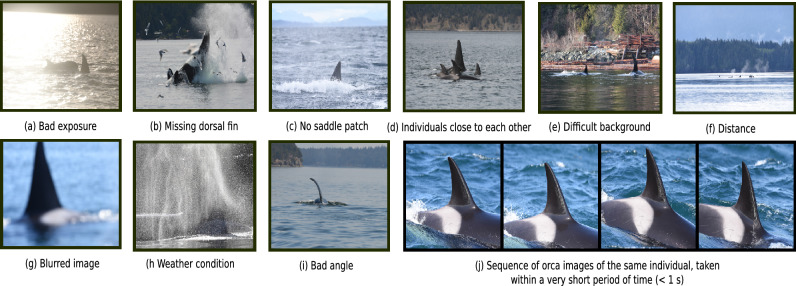
Examples of image content which either lead to completely unusable/invalid data samples, or which make a robust and correct detection/classification much more difficult.

However, the number of usable and correctly labeled images which can actually be utilized for machine learning must be adjusted downward due to several circumstances. Figure 3a–i visualizes multiple examples of situations where images contain valid labels. However, the relevant biometric features are very difficult to recognize or not visible at all. These images cannot be labeled without contextual knowledge, for example by observing previous and/or subsequent images and/or knowing additional information about family-related structures. Therefore, such photos cannot be used for classification of individuals and have to be filtered out out in advance.

Another scenario that impacts the final number of usable identification images is visualized in Fig. 3j. While conducting photo-identification in the field, several images are sometimes taken in very short intervals (< 1 s). However, this procedure leads to several very similar images. To avoid biasing the actual multi-class identification performance by including such images in validation and testing, only the first image of a photo series was machine-selected if the images were taken within a time interval $\delta \le 5\,s$, including the same date and photographer. Considering the photo series visualized in Fig. 3j, only the first image was utilized as a potential sample for network validation or testing. The training material for individual classification was unaffected by this time interval rule, since augmentation procedures change the images during training anyway.

### Killer whale dorsal fin/saddle patch detection (FIN-DETECT)

#### Object detection

In order to extract the regions of interest—killer whale dorsal fin(s) and saddle patch(es)—from the images, an automated and robust object detection has to be conducted. Object detection includes classification and localization of the corresponding object within the respective image^36^. In this context, circumscribing rectangles, so-called bounding boxes, are utilized and drawn around the objects to be recognized. Between a ground truth bounding box and the predicted bounding box, a quality metric named *Intersection over Union (IoU)* ($=\frac{\text {Area of Overlap}}{\text {Area of Union}}$) is often used as a quality criterion^80^.

Two additional evaluation attributes are of essential importance too^36^: (1) *objectness score*—describes the probability that an object is present inside a given bounding box, and (2) *class confidences*—characterize the probability distribution over all distinct object classes. All objects which have to be localized inside an image can strongly vary not only in type and shape, but also in size. Hence, object detection algorithms usually predict a variety of potential bounding boxes. As a result, individual objects may be detected several times by circumscribing bounding boxes, locating at slightly different positions^36^. To counteract this phenomenon, *non-maximum suppression *^36^ *(NMS)* is executed to keep only the best fitting one. Since object detection requires both, correct classification and localization, the metrics per class are determined as follows^81^:

(1) *true positive (TP)*: the target object is within the predicted bounding box area, the bounding box objectness score is larger than a chosen threshold, the object classification and assignment are correct, and IoU between bounding box prediction versus ground truth is higher than a given threshold and all other IoUs of potential overlaying boxes (in case of overlaying boxes, only the box indicating the highest IoU is considered as TP, whereas all remaining boxes are false positives), (2) *false positive (FP)*: the bounding box objectness score is larger than a chosen threshold, but either the target object is not within the predicted circumscribing rectangle, the classification hypothesis is wrong, and/or IoU is smaller compared to any other possible overlaying bounding boxes, (3) *false negative (FN)*: the target object is in the image, but no predicted bounding box hypothesis detected the corresponding object properly, (4) *true negatives (TN)*: object detection ignores TNs, since there are evidently an infinite number of empty boxes with an objectness score that is smaller than a chosen threshold. Based on these traditional binary classification scores, target metrics such as *precision*, *recall*, *F1-score*, *average precision (AP)*, and *mean average precision (mAP)* can be calculated^36^. The average precision describes the *area-under-the-curve (AUC)* of a precision/recall graph, transformed into a monotonically decreasing curve beforehand, calculated on the basis of different IoU thresholds^36^. The AP is calculated for each class, while the mAP refers to the average of all class-related AP scores^36^. Consequently, AP and mAP are identical unless the number of classes is greater than one^36^.

#### Detection data

The dataset which was utilized for training and evaluation of FIN-DETECT was generated via a two-step semi-automatic procedure. In a first step, 2,286 images, originating from various months in 2015, were manually annotated with bounding boxes resulting in the *Human-Annotated Detection Dataset (HADD)*—see Table 1. For this purpose, every dorsal fin and associated saddle patch, visible in each image, were individually circumscribed with a rectangle. FIN-DETECT was trained on HADD using the data distribution reported in Table 1.

The resulting and preliminary version of FIN-DETECT was utilized to automatically apply bounding boxes to randomly chosen unseen images from 2011, 2015, and 2018 in order to enlarge the HADD with machine-identified samples. These samples were not manually verified, but images with no bounding boxes, as well as those with more bounding boxes than labels, were discarded. After applying these rules, a joint dataset, named the *Extended-Annotated Detection Dataset (EADD)*, was created, consisting of the HADD and all valid machine-identified data samples. The resulting EADD (see Table 1) was utilized to retrain FIN-DETECT, which was ultimately applied to all future killer whale detections.

**Table 1:** Human-Annotated Detection Dataset (HADD), including human-labeled dorsal fin/saddle-patch bounding boxes, as well as Extended-Annotated Detection Dataset (EADD) containing human- and machine-labeled dorsal fin/saddle-patch bounding boxes.

Dataset		Split					
		Training		Validation		Test	
		Samples		Samples		Samples	
		Photos	%	Photos	%	Photos	%
HADD^a^	2286	1686	73.8	300	13.1	300	13.1
EADD^b^	7511	5257	70.0	1127	15.0	1127	15.0

^a^*HADD* Human-Annotated Detection Dataset.

^b^*EADD* Extended-Annotated Detection Dataset.

#### Network architecture, data preprocessing, training, and evaluation

FIN-DETECT, visualized in Supplementary Fig. S2, is based on an extended version of the original YOLOv3^76,77^-based object detection architecture. YOLOv3^74–76^ (*You Only Look Once*) is a real-time, single-stage, multi-scale, and fully-convolutional object detection algorithm, which was first introduced as YOLOv1 by Redmon et al.^74^ and continuous improvements have led to the most recent version known as YOLOv5^82^. At the development of FIN-PRINT, YOLOv3 was the most recent version. FIN-DETECT (see Supplementary Fig. S2) essentially consists of two major network parts^74–76,83^: (1) *feature extraction network*, usually referred to as feature extractor and/or backbone network, learning compressed representations (feature maps) of a given input image, representing the foundation for subsequent detection, and (2) *feature pyramid network*, also named head-subnet and/or detector, responsible for detecting objects at three different scales. FIN-DETECT receives as network input a preprocessed, re-scaled, and square $416\,\times \,416$ px RGB-image (zero-padding in case of a none-square original image), resulting in an input shape of $3\,\times \,416\,\times \,416$. The network detects objects utilizing a 13 $\times$ 13, 26 $\times$ 26, and 52 $\times$ 52 grid to recognize large, medium, and small patterns^76,83^ (see Supplementary Fig. S2). FIN-DETECT predicts per cell a $1\,\times \,21$ detection vector, which contains $b=3$ different bounding boxes and $c=2$ classes (dorsal fin/saddle patch vs. no dorsal fin/saddle patch), combined with four 0/1-normalized bounding box coordinates (*x*, *y*, *w*, *h*) and one objectness score per box, resulting in b $*$ (5 $+$ c) $=$ 21 elements per cell. Consequently, the scale-dependent detection outputs of FIN-DETECT comprised a final output shape of $13\,\times \,13\,\times \,21$, $26\,\times \,26,\times \,21$, and $52\,\times \,52,\times \,21$ (see Supplementary Fig. S2). More detailed information about YOLO in general, YOLOv3, and/or other YOLO versions can be found here^74–76,82,84,85^.

The backbone network (Darknet-53^76^) of FIN-DETECT was initialized with pre-trained weights on ImageNet^86^. A detailed overview about all other network hyperparameters is given in Supplementary Table S2. Moreover, FIN-DETECT implements the following YOLOv3^76^ detection parameters: objectness score threshold of 0.5 (training, validation) and 0.8 (testing), IoU threshold of 0.5, and NMS threshold equals to 0.5. FIN-DETECT reports precision, recall, F1-Score, and mean average precision as evaluation metrics. Based on a given input image, FIN-DETECT returns a text file containing 0/1-normalized bounding box information (*x*, *y*, *w*, *h*) of every detection hypothesis.

### Killer whale dorsal fin/saddle patch extraction (FIN-EXTRACT)

FIN-EXTRACT facilitates automatic extraction and subsequent rescaling of previously detected and marked image sub-regions using the bounding box information derived by FIN-DETECT. For each identified bounding box, a square $512\,\times \,512$ px RGB-sub-image was cropped from the original photo. In a first step, the 0/1-normalized bounding box information (*x*, *y*, *w*, *h*) was multiplied by the original image shape to obtain the correct coordinates within the original image. In case a bounding box was not square, the larger of the two dimensions was utilized to reshape the original detection rectangle. Furthermore, it was verified whether a bounding box extended beyond the edge of an image and moved accordingly if necessary. In case the original image was smaller than $512\,\times \,512$ px, it was interpolated and resized respectively. Otherwise, a sub-image, based on the original bounding box size, was cropped and if applicable compressed and resized to $512\,\times \,512$ px. Depending on the resized bounding box, this may result in a bit more background content. However, any kind of zero-padding is avoided for subsequent individual classification. In addition, the image quality of the final extracted sub-image(s) depends on the original image resolution, along with the distance of the individual(s) within the captured photos.

### Valid versus invalid (VVI) dorsal fin/saddle patch detection (VVI-DETECT)

#### VVI detection

Considering potential detection errors (e.g. tail and/or pectoral fins, triangular formed head of the animal, etc.), besides all the different challenging situations visualized in Fig. 3a–i, additional data enhancement is indispensable (see also examples in Supplementary Fig. S3). All these scenarios either result in completely unusable/invalid (e.g. missing dorsal fin, no saddle patch, bad angle, distance, detection errors), or insufficient quality images (e.g. poor weather conditions, bad exposure, blurred image). Without sufficient domain knowledge and additional meta-information (e.g. images shortly taken before, other animals in the image, family-related structures, etc.), all the aforementioned situations lead to invalid identification images which are not able to be classified correctly by human or machine. Detected/extracted RGB-sub-images containing a single dorsal fin and saddle patch are considered as valid identification images. To filter the majority of such invalid samples originating from previous processing levels, a binary classification network was designed to distinguish between two classes—*Valid Versus Invalid* (VVI)—killer whale identification images prior to final multi-class individual recognition. Supplementary Fig. S3 visualizes some of the challenging pre-detected/-extracted sub-images, belonging to the *invalid* class.

#### Detection data

In order to train VVI-DETECT, a two-class dataset, named *Valid/Invalid Killer Whale Identification Dataset 2011–2017 (VIKWID11-17)*, was utilized. Table 2 describes VIKWID11-17 in combination with the respective data distribution. VIKWID11-17 is a manually labeled data archive based on randomly chosen, previously detected (FIN-DETECT), and extracted (FIN-EXTRACT) sub-images from 2011 to 2017. In addition to multiple valid pre-detected/-extracted identification images of different individuals, the dataset also includes examples of invalid sub-images covering the scenarios illustrated in Fig. 3a–i. Furthermore, the invalid class was extended by examples of images with potential detection errors (noise), such as water, boats, coastline, houses and/or other landscape backgrounds, to also filter such cases in advance. During data selection an interval of 5 s was applied to the validation and test set (see Fig. 3j) in order to not distort classification accuracy in any way.

**Table 2:** Valid/Invalid Killer Whale Identification Dataset 2011–2017 (VIKWID11-17), a human-annotated dataset consisting of valid and invalid identification images (dorsal fin + saddle-patch), utilized to train, validate, and test VVI-DETECT, after applying the interval rule of 5 s with respect to the validation and test set.

Dataset		Split											
		Training				Validation				Test			
		Samples				Samples				Samples			
		Valid	Invalid	$\sum$	%	Valid	Invalid	$\sum$	%	Valid	Invalid	$\sum$	%
VIKWID11-17^a^	1590	700	509	1209	76.0	126	89	215	13.5	83	83	166	10.5

^a^*VIKWID11-17* Valid/Invalid Killer Whale Identification Dataset 2011–2017.

#### Network architecture, data preprocessing, training, and evaluation

VVI-DETECT, visualized in Supplementary Fig. S3, is a ResNet34^78^-based convolutional neural network (CNN), designed for binary classification between valid versus invalid (VVI) identification images. Residual networks^78^ (ResNets) consist of a sequence of residual layers, which are built up from building blocks including concatenations of weight (e.g. convolutional/fully-connected), normalization (e.g. batch-norm^87^), and activation layers (e.g. ReLU^88^), together with residual-/skip-connections^78^. These connections allow the network to optimize a residual mapping $F(x)\,=\,H(x)\,-\,x$ with respect to a given input *x*, rather than directly learning an underlying mapping *H*(*x*)^78^. This type of learning, called residual learning, opens up the possibility to train deeper models^78^. The use of different building block types, together with the number of blocks, results in various ResNet architectures, like ResNet18, ResNet34, ResNet50, ResNet101, and ResNet152^78^. For more detailed information about the concept of residual learning/networks, see He et al.^78^. Compared to the original ResNet34 architecture, the size of the initial $7\,\times \,7$ convolution kernel was changed to $9\,\times \,9$, in order to cover larger receptive fields at the initial stage. As network input, VVI-DETECT receives data of previously detected (FIN-DETECT) and extracted/reshaped (FIN-EXTRACT) $3\,\times \,512\,\times \,512$-large RGB-pictures for both classes. The network output is a $1\,\times \,2$ probability vector, containing class-wise model prediction probabilities (see Supplementary Fig. S3). Based on preliminary investigations, ResNet34^78^ proved to be the most efficient version for this entire study in terms of performance and computation efficiency compared to other ResNet architectures. VVI-DETECT integrates an augmentation procedure consisting of eight different functions: (1) addition of random Gaussian noise to the image, (2) image rotation at maximum angle of ± 25 degree, (3) blurring the image by applying a gaussian blur, (4) mirroring the picture with respect to the y-axis, (5) edge enhancement within the image, (6) sharpening the input picture, (7) brighten/darken of the image, and (8) random color change by swapping the RGB channels. Out of this function pool, number, type, and arrangement of augmentation operations were randomly determined for each image within the training phase (no augmentation during validation and testing). The random number of augmentations per image was within an interval of $[1\,:\,a_{max}]$ with $a_{max}\,\in \,[1\,:8\,]$ being constant across the entire training. In this study the maximum augmentation number per image was set to $a_{max}=5$. VVI-DETECT reports accuracy, precision, recall, F1-Score, and false-positive-rate. A detailed description of all relevant network hyperparameters is illustrated in Supplementary Table S2.

### Individual killer whale classification network (FIN-IDENTIFY)

#### Individual killer whale classification

Robust multi-class killer whale individual classification requires representative and high-quality animal-specific image data in sufficient quantity. However, significant variations can be observed in the total number of animal-specific images (see Fig. 2). In addition, multiple and essential data constraints have been introduced which strongly affect the actual amount of usable identification images per individual, such as (1) only single-labeled images together with exactly one predicted bounding box hypothesis, (2) data enhancement by pre-filtering invalid identification images to avoid situations visualized in Fig. 3a–i, and (3) time interval rule of 5 s during network validation and testing to counteract the effect of classifying very similar photos, visualized in Fig. 3j. Moreover, all photos from 2018 were completely ignored for additional network evaluation purposes. Additionally, all images including more than a single label (in total 34,306 pictures, 2011–2018, see Fig. 2) could not be used for training an initial multi-class identification network due to the *label assignment problem*. The *label assignment problem* describes the situation where an image contains multiple individuals and labels, however, it is unknown which label belongs to which individual. All these data restrictions and constraints led to a significant, qualitative improvement of the material, but also considerably reduced the amount of usable data. In summary, these data limitations led to a final representation of the 100 (out of 367) most commonly single-labeled Bigg’s individuals (see Fig. 2), present across all years (2011–2018), representing about 64% (55,305 photos) of the entire single annotated and original data from 2011 to 2018 (86,789 images). Based on the top-100 killer whales, the smallest individual-specific number of remaining data samples comprised 135 images (see Table 3), to still provide sufficient variation and data diversity combined with various image augmentation techniques during model training. Despite previous filtering by VVI-DETECT and to avoid potential errors caused by previous processing levels, the proposed *invalid* class was also included at this stage resulting in a final 101-class (100 individuals, 1 rejection class) procedure.

#### Identification data

FIN-IDENTIFY was trained on two different datasets, both illustrated in Table 3. The first dataset, named *Killer Whale Individual Dataset 2011–2017 (KWID11-17)*, consisted of 39,464 excerpts including only a single label, distributed across 101 classes, and recorded between 2011 and 2017 (see Table 3). All excerpts were machine-annotated, applying FIN-DETECT, FIN-EXTRACT, and VVI-DETECT in a sequential order, following the previously mentioned data constraints and restrictions. VVI-DETECT considered an image to be invalid if the network confidence was p$_{invalid}$ > 0.85. The VIKWID11-17 dataset (see Table 2), on which VVI-DETECT was trained on, is completely independent from the entire data listed in Table 3. KWID11-17 consists of 36,457 images being assigned to the valid class, whereas 3007 photos were added to the invalid class, representing a small portion of the overall amount of detected invalid images across 2011 to 2017 in order to not bias class distributions. Table 3 presents the final data distribution of KWID11-17 as well as dataset-specific statistics.

To add additional data and simultaneously counteract the *label assignment problem*, the first version of FIN-IDENTIFY, trained on KWID11-17, was applied to all images from 2011 until 2017, including those with multiple labels and either one or more of the trained 100 individuals. FIN-IDENTIFY classified all potential detected (FIN-DETECT) and extracted (FIN-EXTRACT) labels for each image containing more than one animal. If the best classification hypothesis (class with the highest probability) per sub-image matches one of the original labels applied to that image, it was considered as correctly classified and added to the respective class. The resulting extended dataset, entitled *Killer Whale Individual Dataset Extended 2011–2017 (KWIDE11-17)*, together with the corresponding data distribution, was utilized to train an updated and more robust version of FIN-IDENTIY (see Table 3). KWIDE11-17 consists of KWID11-17, extended by the additional machine-identified multi-label material, leading to a total number of 65,713 excerpts, distributed across 101 classes. The total number of valid identification images is 62,740, whereas the invalid class comprises 2,973 images. KWID11-17 and KWIDE11-17 use the same portion of machine-annotated invalid data excerpts, however, the overall number of samples slightly differs (KWID11-17—3007 versus KWIDE11-17—2973) due to a different split, in combination with the applied interval rule of 5 s during validation and testing.

**Table 3:** Killer Whale Individual Dataset 2011–2017 (KWID11-17), including machine-annotated data of valid images (dorsal fin $+$ saddle-patch) for the 100 most commonly photographed individuals satisfying the data constraints (one label per image $+$ exactly one bounding box prediction), in combination with machine-annotated invalid data utilizing VVI-DETECT after applying the interval rule of 5 s.

Dataset		Split											
		Training				Validation				Test			
		Samples				Samples				Samples			
		Valid	Invalid	$\sum$	%	Valid	Invalid	$\sum$	%	Valid	Invalid	$\sum$	%
KWID11-17^a^	39,464	27,238	2227	29,465	74.7	4940	395	5,335	13.5	4279	385	4664	11.8
KWIDE11-17^b^	65,713	48,200	2226	50,426	76.7	7729	392	8121	12.4	6811	355	7166	10.9

Killer Whale Individual Dataset Extended 2011–2017 (KWIDE11-17) extends the KWID11-17 data archive with images of the 100 most common individuals represented in images containing more than one label and classified via the first version of FIN-IDENTIFY, trained on KWID11-17. Notice that the distribution of the invalid photos differs slightly between KWID11-17 and KWIDE11-17 due to the different data splits and subsequent effect of the 5 s interval rule. Furthermore, additional statistics regarding the number of identification images (100 classes) are reported for both datasets.

^a^*KWID11-17* Killer Whale Individual Dataset 2011–2017—Statistics on the number of identification images (100 most common classes):

mean = 364.57, stdv = 162.91, min = 135 (T073B), max = 916 (T019B)

training stats (only valid images): mean = 272.38, stdv = 125.79, min = 107 (T073B), max = 695 (T019B)

validation stats (only valid images): mean = 49.40, stdv = 20.81, min = 16 (T073B), max = 120 (T019B)

testing stats (only valid images): mean = 42.79, stdv = 18.60, min = 8 (T121A), max = 101 (T019B).

^b^*KWIDE11-17* Killer Whale Individual Dataset Extended 2011–2017—Statistics on the number of identification images (100 most common classes): mean = 627.40, stdv = 245.06, min = 172 (T073B), max = 1442 (T019B)

training stats (only valid images): mean = 482.00, stdv = 192.32, min = 139 (T073B), max = 1122 (T019B)

validation stats (only valid images): mean = 77.29, stdv = 28.74, min = 17 (T073B), max = 174 (T019B)

testing stats (only valid images): mean = 68.11, stdv = 26.98, min = 10 (T121A), max = 146 (T019B).

#### Network architecture, data preprocessing, training, and evaluation

FIN-IDENTIFY, visualized in Supplementary Fig. S4, is a ResNet34^78^-based convolutional neural network (CNN), created for multi-class individual classification. The network architecture is identical to VVI-DETECT (see Supplementary Fig. S3) except for the final 101-class output layer ($1\,\times \,101$ probability vector). FIN-IDENTIFY was trained on the $3\,\times \,512\,\times \,512$ sub-images, generated by FIN-EXTRACT and if necessary filtered by VVI-DETECT (see Fig. 1 and Supplementary Fig. S4). Besides the same network architecture, identical interval rule conditions (5 s) were applied during training. Data augmentation and preprocessing was also identical to VVI-DETECT and all other required network hyperparameters are listed in Supplementary Table S2. Next to the overall accuracy, FIN-IDENTIFY reports a top-3 weighted (TWA) and unweighted accuracy (TUA). TWA describes whether the target class probability is within the top-3 and if so, a rank-dependent weight is assigned ($\omega _{1}\,=\,1$, $\omega _{2}\,=\,0.5$, and $\omega _{3}\,=\,0.25$). TUA illustrates, if the target individual is within the top-3, it is counted as correct, independent of the respective rank. For both metrics, either the sum of all weighted, or correct predictions is divided by the total number of classifications.

## Experiments

The following major experiments were conducted: (1) training/evaluating FIN-DETECT on the dataset listed in Table 1 (HADD, EADD), to derive a robust dorsal fin/saddle patch detection network, (2) training/evaluating VVI-DETECT on the data (VIKWID11-17) presented in Table 2, (3) training/evaluating FIN-IDENTIFY with respect to the datasets (KWID11-17, KWIDE11-17) reported in Table 3, and (4) applying the entire FIN-PRINT pipeline (see Fig. 1), while utilizing the best previously trained networks, to all original, unseen, and single-labeled images from 2018, containing individuals which are modeled and represented within the 100 classes of FIN-IDENTIFY (see Supplementary Table S1).

## Results

### FIN-DETECT and FIN-EXTRACT

**Figure 4:**
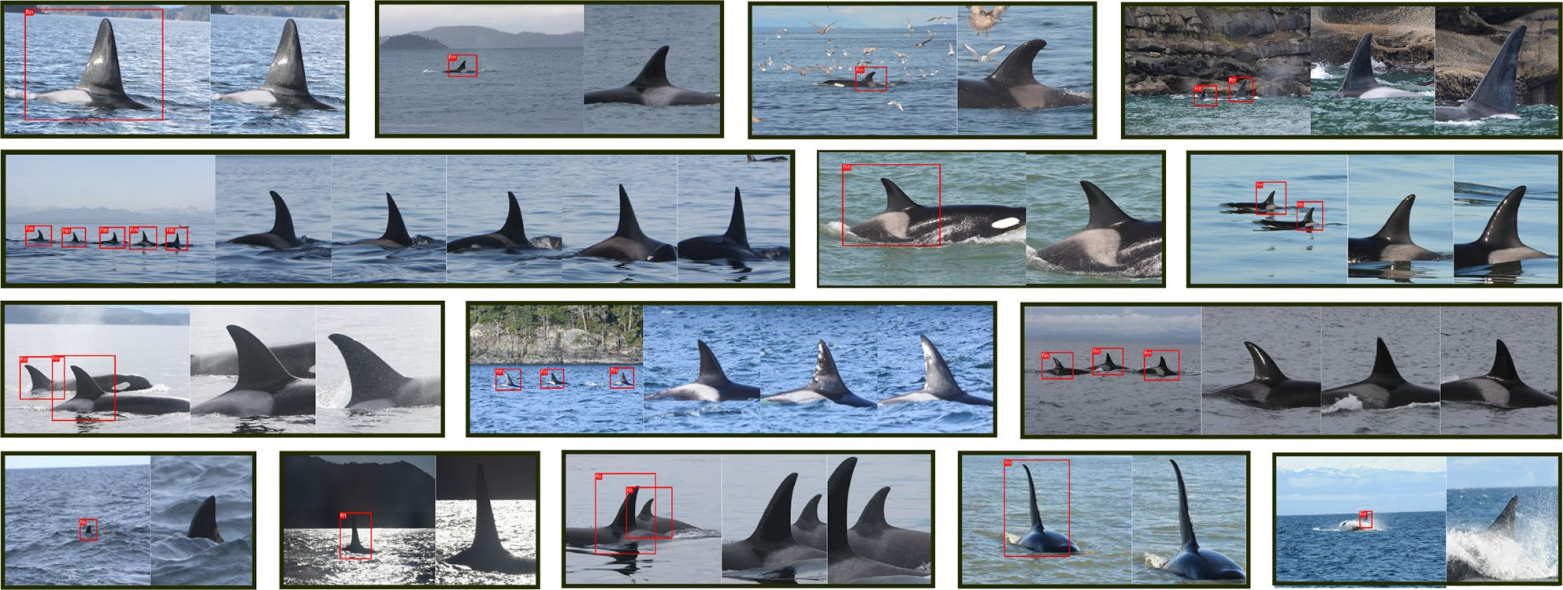
Dorsal fin/saddle patch detection and extraction results based on randomly chosen identification images from various years (2011–2017), applying FIN-DETECT, trained on the machine-extended EADD data archive (see Table 1), and FIN-EXTRACT.

Table 4 reports validation and test results (recall, precision, F1-score, mAP) of FIN-DETECT evaluated on both detection datasets—HADD and EADD (see Table 1). Despite the fact that both data archives are not directly comparable, because of different data volumes and distributions, the automated and machine-driven data enlargement shows significant improvements with respect to the validation and test metrics. The version of FIN-DETECT trained on the EADD data material was utilized within all subsequent machine detection tasks. In addition to the traditional object recognition metrics listed in Table 4, various detection and extraction examples have been visualized in Fig. 4. All detection results, visualized in Fig. 4, were computed by applying FIN-DETECT, trained on the machine-extended EADD, to some random and unseen images from different years. Next to the detected and valid identification sub-images, represented by the red circumscribing bounding boxes (see Fig. 4), the associated extractions were created applying FIN-EXTRACT, together with the corresponding bounding box information. The image pairs, visualized in Fig. 4, consist of detection results and corresponding extractions. Besides valid fin/saddle patch detection results, example images of invalid, but correctly detected identification images, are displayed as well (see Fig. 4, last row). In all these cases the dorsal fin was detected correctly, however, due to lack of information and/or very challenging scenarios, the extracted sub-images are unusable/invalid for future individual identification (bad angle, no saddle patch, individuals close to each other, bad exposure, difficult background—see also Fig. 3a–i).

**Table 4:** Detection results while training two versions of FIN-DETECT with respect to HADD and EADD.

Metric	Dataset			
	HADD^a^		EADD^b^	
	Validation [%]	Test [%]	Validation [%]	Test [%]
Recall	95.0	89.0	95.1	94.4
Precision	80.0	85.0	94.2	94.1
F1-Score	86.9	87.0	94.7	94.2
mAP^c^	92.0	82.0	94.2	93.4

^a^*HADD* Human-Annotated Detection Dataset.

^b^*EADD* Extended-Annotated Detection Dataset.

^c^*mAP* mean Average Precision.

### VVI-DETECT

**Figure 5:**
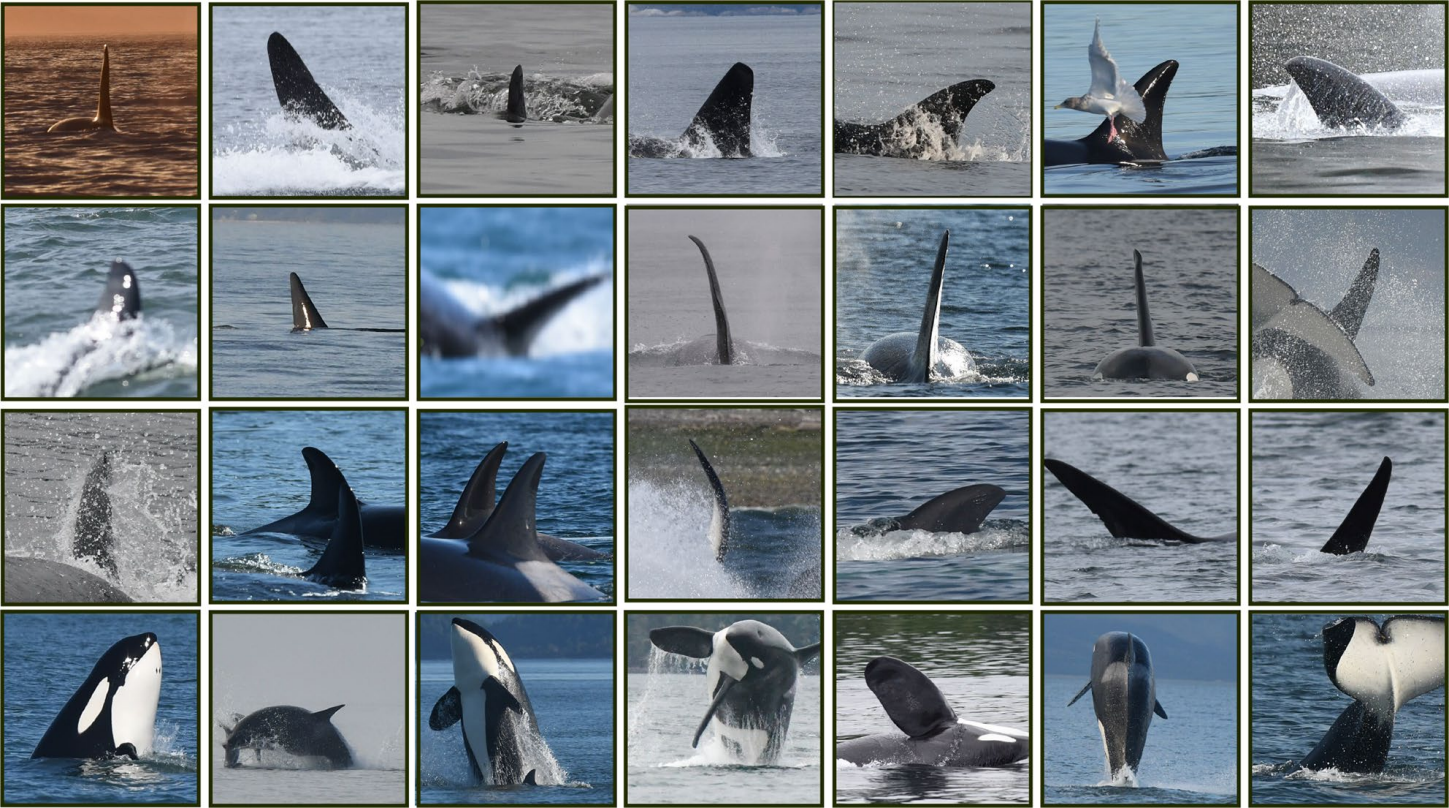
Detected (FIN-DETECT) and extracted (FIN-EXTRACT) unseen identification images from 2018, which were successfully categorized and filtered as invalid identification images by VVI-DETECT, trained on VIKWID11-17, reported in Table 2.

Table 5 reports validation and test results of VVI-DETECT evaluated on VIKWID11-17 (see Table 2). This model was utilized for all required valid versus invalid image predictions. Besides validation and test metrics, example images of various, correctly predicted and filtered invalid identification photos from the unseen 2018 dataset are visualized in Fig. 5. The sub-images, presented in Fig. 5, reflect the previously mentioned variety of challenging scenarios shown in Fig. 3a–i.

**Table 5:** Detection results of VVI-DETECT to filter between valid versus invalid identification images (data enhancement), while training VVI-DETECT on VIKWID11-17.

Metric	Dataset	
	VIKWID11-17^a^	
	Validation [%]	Test [%]
Recall	97.8	92.8
Precision	92.6	97.5
FPR	5.6	2.4
F1-Score	95.1	95.1
Accuracy	95.8	95.2

^a^*VIKWID11-17* Valid/Invalid Killer Whale Identification Dataset 2011–2017.

The photos from 2018 that are shown in Fig. 5 visualize examples of invalid identification images due to poor image quality (lighting, exposure, etc) or poor subject representation (bad angle, too distant, dorsal fin and saddle patch not shown, etc.) (see also Fig. 3a–i). The problem regarding such detection errors is that at least one appendage (tail, pectoral, and/or dorsal fin) is present in most of these images (see Fig. 5, detection errors—last row). Furthermore, there are also cases where the shape of the recognized object is very close to the triangular structure of the fin (e.g. a spyhop where the killer whale lifts its head out of the water, see last row in Fig. 5). All these invalid data samples were successfully pre-filtered utilizing VVI-DETECT as an additional data enhancement step, to avoid subsequent misclassifications during final individual recognition (see FIN-IDENTIFY).

### FIN-IDENTIFY

The last step of the entire FIN-PRINT pipeline, visualized in Fig. 1, describes final individual multi-class identification. Due to reasons of comparison, the results for both models—the preliminary version and the final FIN-IDENTIFY network—are reported. In both cases the overall 101-class accuracy, the top-3 weighted (TWA) and unweighted (TUA) accuracy, is presented on the validation and test set, all together visualized in Table 6. Both FIN-IDENTIFY models show similar validation and test metrics, which thus provide no evidence of overfitting. Although both datasets (KWID11-17 and KWIDE11-17) are not comparable due to different splits and distributions, the additional machine-annotated images of the 100 most common individuals result in a significant improvement in model performance, generalization, and transferability. For all pending unseen classification events, FIN-IDENTIFY trained/evaluated on KWIDE11-17, was applied. Moreover, such consistently promising multi-class classification results prove feasibility and quality of the entire FIN-PRINT pipeline (see Fig. 1).

**Table 6:** Individual killer whale classification results (101-classes), while training two versions of FIN-IDENTIFY, using the initial KWID11-17 or KWIDE11-17 datasets.

Metric	Dataset			
	KWID11-17^a^		KIWIDE11-17^b^	
	Validation [%]	Test [%]	Validation [%]	Test [%]
Accuracy	85.8	86.7	91.1	92.5
TWA^c^	89.0	89.9	93.4	94.6
TUA^d^	93.2	94.3	96.3	97.2

^a^*KWID11-17* Killer Whale Individual Dataset 2011–2017.

^b^*KWIDE11-17* Killer Whale Individual Dataset Extended 2011–2017.

^c^*TWA* Top-3 Weighted Accuracy.

^d^*TUA* Top-3 Unweighted Accuracy.

### FIN-PRINT—Unseen Year 2018

To further verify performance and generalization, the entire FIN-PRINT pipeline (see Fig. 1) was applied to unseen data from 2018. The best FIN-DETECT, VVI-DETECT, and FIN-IDENTIFY model was applied in a sequential order (see FIN-PRINT workflow in Fig. 1) to predict identification labels for the 100 most commonly photographed individuals, being covered by FIN-IDENTIFY. All single-labeled images in the 2018 dataset, which include one of these 100 individuals, were automatically processed by FIN-PRINT (detection, extraction, filtering, and classification—see Fig. 1). A total of 5,768 single-labeled sub-images, each of them belonging to one of the 100 most commonly photographed animals, were detected and extracted applying FIN-DETECT/-EXTRACT, while considering the previous data constraint of a single label together with exactly one bounding box. Afterwards, VVI-DETECT was applied to pre-filter the 5,768 identification images, which machine-identified 1057, either challenging, and/or unusable/invalid excerpts (see Fig. 3a–i) resulting in 4711 valid identification sub-images of the 100 most commonly photographed individuals. On average, each animal occurred 47.1 times, with a standard deviation of 30.2. In the 2018 dataset, *T109* was the least photographed individual with only 2 images, whereas *T100C* was the most frequently photographed with 132 identification images. Finally, FIN-IDENTIFY, trained on KWID11-17 and KWIDE11-17, was applied to predict the respective identification labels. Within a real-world scenario, one would not need to continue looking at the previously machine-filtered 1057 invalid material in case of individual classification and directly process the remaining 4711 valid samples. However, to demonstrate and prove the necessity of introducing a rejection class also at the final stage of individual classification (FIN-IDENTIFY), all 5768 unseen images were used for prediction. Furthermore, in practice, only the final version of FIN-IDENTIFY, trained on KWIDE11-17, would be applied to unseen data.

**Figure 6:**
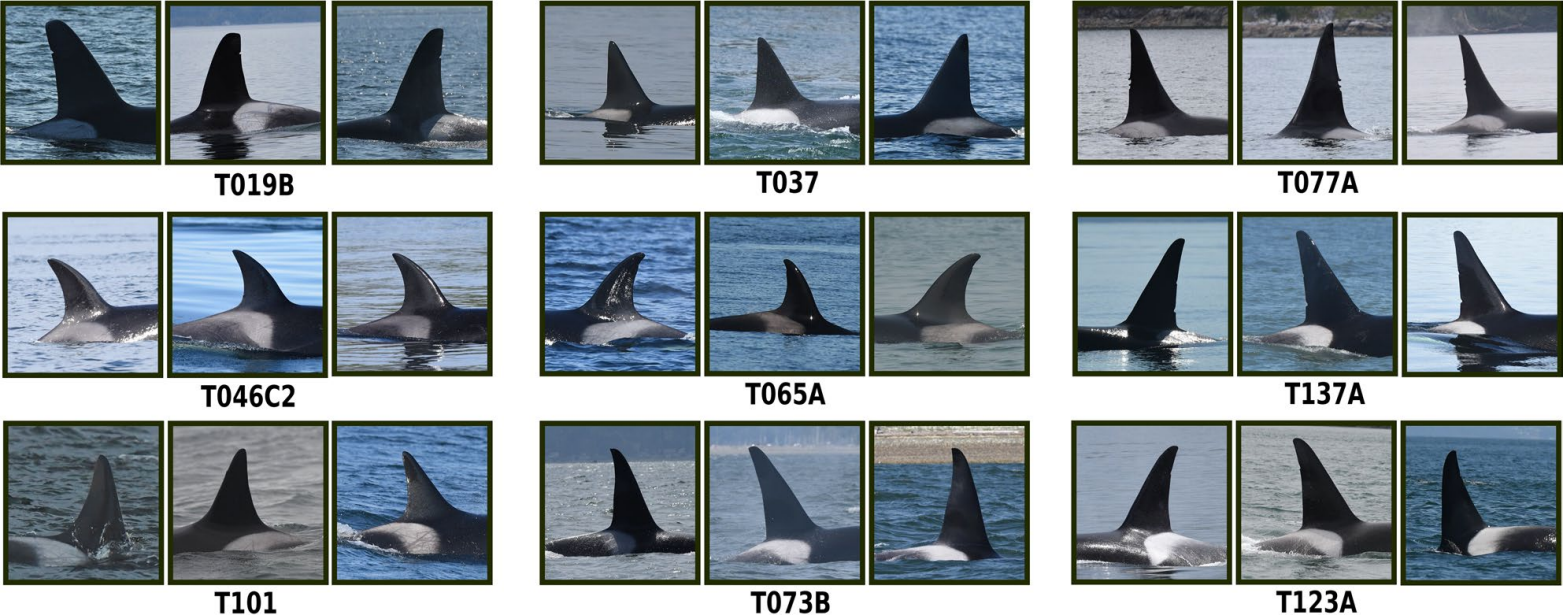
Examples of detected (FIN-DETECT), extracted (FIN-EXTRACT), and pre-filtered (VVI-DETECT) unseen and valid killer whale identification images from 2018 which were successfully classified by FIN-IDENTIFY, trained on KWIDE11-17.

FIN-IDENTIFY, trained on KWID11-17, achieved an accuracy of 82.8%, next to a top-3 weighted and unweighted accuracy of 86.6%, as well as 91.7%, based on the 101-class task. Training FIN-IDENTIFY on KWIDE11-17, resulted in an accuracy of 84.5%, next to a top-3 weighted and unweighted accuracy of 88.1%, as well as 92.9%. Figure 6 visualizes correct classification examples of the extended classifier version for 9 individual killer whales from the unseen data from 2018.

## Discussion

The current study presents a fully machine-based, multi-stage, deep-learning pipeline, named FIN-PRINT (see Fig. 1), with the aim to automatize and support the analysis of killer whale photo-identification data. Dorsal fin and saddle patch detection, the first step of FIN-PRINT, was performed via a two-stage training procedure. The initial version of FIN-DETECT achieved promising results (see Table 4), hence additional machine-annotated data was generated by applying the model to unseen data from 2011, 2015, and 2018 (see Table 1). Whereas validation and test results on the smaller HADD dataset slightly diverge, they both significantly and consistently improved while training/evaluating FIN-DETECT on the machine-extended EADD (see Table 4). However, a direct comparison between both models is difficult because the volume and distribution of data were different (see Table 1). Based on the detected bounding box coordinates, equally-sized $512\,\times \,512$ RGB-sub-images were extracted and if necessary interpolated or compressed (no zero-padding), using FIN-EXTRACT, the second step of FIN-PRINT. However, the quality of detected and extracted sub-images is not solely dependent on the performance of FIN-DETECT, but also on the original image content and quality (see Figs. 3 and 5 ).

Most of these images contain dorsal fins, leading to correct identifications by FIN-DETECT, however they are useless for downstream individual classification. Besides these cases, images of other body parts, such as tail flukes, pectoral flippers, or other triangular structures (e.g. head of a killer whale), often exist. Such false detections do have strong similarities, hence making them difficult to avoid. Consequently, it is imperative to conduct a data enhancement procedure to filter such invalid identification images beforehand. For this reason, VVI-DETECT, the third step of FIN-PRINT, was trained and evaluated on the manually labeled VIKWID11-17 (see Table 2). Binary classification metrics of VVI-DETECT on the unseen test set (see Table 5) provide no indication of overfitting. In addition, several examples of invalid pre-detected/-extracted identification images, correctly identified by VVI-DETECT, are visualized in Fig. 5, representing all the challenging situations previously described in Fig. 3a–i and clearly proving the enormous importance of such a preliminary data enhancement procedure. The final step of FIN-PRINT—killer whale individual classification—was conducted in a two-step process, similar to FIN-DETECT. First, a preliminary version was trained and evaluated on KWID11-17 (see Table 3), showing no evidence of overfitting. The top-3 classification hypothesis (TWA/TUA) greatly improves the chance of observing the correct prediction, while simultaneously reducing the dimensionality of potentially eligible individuals by an order of magnitude (101 versus 3 classes).

The final version of FIN-IDENTIFY was trained and verified on KWIDE11-17, whereby the overall classification performance was significantly improved by the data expansion (86.7% versus 92.5%) and no sign of overfitting was observed. A 5.8% increase in accuracy results in an error reduction rate of 43.6%. Considering the difference of 2.9% regarding the top-3 unweighted accuracy (94.3% versus 97.2%) an error reduction rate of 50.9% was achieved. Due to different data volumes and distributions, results of the preliminary and final model (see Table 6) cannot be directly compared. However, the consistent improvements on validation and test are a good indication for a working FIN-PRINT pipeline.

Despite all the promising dataset-specific results, an additional real-world evaluation scenario was simulated. Identification image data are typically labeled at the end of an annual fieldwork period. While considering such a procedure, the year 2018 was disregarded, to provide FIN-PRINT with new and unseen data. Due to evaluation purposes, the number of images in 2018 was limited to only those containing the 100 most common individuals. Moreover, only single-labeled identification images, together with exactly one bounding box hypothesis, were analyzed. Contrary to the previous changing datasets, a direct comparison of the classification models is now possible. Within this real-world evaluation scenario the performance of both 101-class classifiers clearly shows a working FIN-PRINT pipeline. Furthermore, a significant performance improvement is shown in the analysis of the 2018 dataset, with respect to the dataset the classifier was trained on. An accuracy difference of 1.7% (82.8.% versus 84.5%) led to an error reduction of 9.9%, whereas a TUA difference of 1.2% (91.7% versus 92.9%) resulted in an error reduction rate of 14.5%. Considering how fine details in the appearance of individuals change naturally over time, in combination with completely different environmental conditions (weather, water, background, and/or changing cameras), the results are very promising.

A one-to-one comparison with results from other machine-learning studies identifying individuals proved to be very difficult due to: (1) different species and use-cases, (2) variability in datasets (amount of data, type of annotations, labeling granularity, data distribution, etc.), (3) completely different or slightly deviating approaches, and (4) varying evaluation scales and metrics. However, to emphasize and clearly demonstrate the value of this work, FIN-PRINT was compared to the most recent studies and state-of-the-art concepts addressing detection and classification of individuals represented in image data.

Animal localization and classification (object detection) are often modeled within a single network (e.g. YOLO^74–77^) at the same time^67^. Such an approach is not recommended for the identification of individuals belonging to a certain species, as it can cause significant reduction in the system’s robustness. On the one hand, there is no possibility to filter out potential object localization errors by subsequent algorithms. On the other hand, the joint feature representations, learned for localization and classification, generally prove not to be ideal especially when distinguishing very similar objects, as is the case when recognizing individuals within a species, rather than cross-species recognition.

Recent studies also apply approaches such as posture identification^38,40^ to incorporate additional information. Moreover, alignment points (landmarks) are frequently used^43,45,48,54,55^ to adjust, orientate, and standardize images regarding their final alignment to receive homogeneous data samples and consequently counteract the scale and rotation invariance of CNNs. In case of killer whale individual identification, such concepts are not relevant. Images are taken from either the left and/or right side of the animal’s body as soon as they surface to fully identify both, fin and saddle patch. These body features are often the only ones visible as well as the only ones necessary for identification (see Fig. 6). Images where the fin and saddle patch are hidden and/or not sufficiently visible because of a poor angle (see Fig. 3i and examples in Fig. 5) can not be used, even after rotation, making an alignment procedure superfluous.

Several recent methods designed for automated image identification were evaluated on considerably smaller and less complex datasets^38,39,42,43,50,51,54,59,61,64,68,69^, shorter time series datasets^50,59^, and data collected from geographically limited locations^50,54,55,59^. FIN-PRINT, however, was analyzed on a large-scale dataset (roughly 121,000 images of 367 individuals), collected over 8 years within a huge territory. This introduced complexity to the dataset, which was intensified through changing killer whale markings over time.

The work of Thompson et al.^64^ is to some extent a similar study, which includes several sequentially-ordered steps to automate and expedite the individual recognition of common bottlenose dolphins (*Tursiops truncatus*). It must be considered that for common bottlenose dolphins only the fin is used as identification criterion, whereas killer whales also have the saddle patch. However, the system achieved a top-ranked accuracy of 88.1%, top-10 of 93.6% and top-50% of 97.0%, evaluated on 672 images and 420 unique animals. FIN-PRINT, by comparison, achieved 97.2% top-3 accuracy on the unseen test data (7166 images, 100 animals), as well as 92.9% top-3 accuracy on the entire and unseen year 2018 (5768 images, 100 animals).

Data distribution is also very important next to the mentioned data complexity. Most of the research approaches did not have uniformly distributed image data for each individual^42,48,55,61^, which means that some animals are observed significantly more often than others, leading to the aforementioned long-tailed distribution. Exactly the same long-tailed phenomenon can be observed in our case (see Fig. 2), which strongly affects the number of killer whales being represented within the final classification model due to a limited number of training samples. In order to address these problems, most studies either use traditional classifiers^42,48^ (e.g. SVM), which do not require such large data volumes compared to deep learning methods, but usually also provide worse classification results, or apply Deep Metric Learning^38,40,45,46,49,51,52,61,64^, especially in combination with the triplet loss^71–73^. Considering the aforementioned difficulties regarding the initial usage of the triplet loss and identification of appropriate triplets, traditional supervised classification was performed as an initial step. However, together with FIN-IDENTIFY, it is now possible to automatically generate appropriate hard and semi-hard triplets^73^ for 100 individuals, based on the top-N classification hypothesis. Thus, robust and efficient Deep Metric Learning will be possible in the future, allowing an extension to all 367 individuals, regardless of the number of images per killer whale, which consequently also solves the previously mentioned problem regarding the long-tailed data distribution. In addition, it is not necessary to retrain the classification system in case new animals have to be added.

Robust representation learning is essential for final classification. Hu et al.^89^ introduced an impressive representation learning approach for multi-label images applying a Graph Attention Network (RRL-GAT). Results on two well-known image datasets have shown significant performance improvements compared to all current state-of-the-art methods^89^. This promising approach could benefit even further from the strong limitations of potential objects/labels present in killer whale identification images, which in turn could improve the focus on interesting image regions, all of which will be the task of future research activities.

Due to the promising accuracy, together with a high performance during inference, FIN-PRINT will be the key element of an interactive web-based server/client labeling system in the future, supporting biologists during their daily work (data maintenance and analysis). In addition, it will also be possible for anyone to access and upload killer whale images worldwide via a web interface. Consequently, FIN-PRINT must be able to process images of widely varying quality (different cameras, locations, photographers, environmental conditions, etc.) as accurately as possible, making a deep learning-based quality inspection (VVI-DETECT) indispensable. Thus, FIN-PRINT facilitates efficient and robust processing of large volumes of killer whale photo-identification data. The overall classification accuracy as well as efficient response time during network inference allow FIN-PRINT to be used in conjunction with video recordings for real-time detection and classification, as well as offline evaluation of the recorded video footage.

Future work will also include artificial data enlargement to counteract the mentioned long- tailed data distribution phenomenon and accompanying data sparsity for most of the individuals in the population (see Fig. 2). For this purpose, deep learning-based algorithms in connection with 3D-modeling approaches will be examined. Besides data augmentation techniques, additional investigations will be conducted to counteract current data limitations visualized in Fig. 3. In the context of this study, photos with bad weather conditions, next to originally blurred images (see Fig. 3g,h), and/or vague examples caused through the magnification of detected and extracted distant dorsal fins (see Fig. 3f), were machine-filtered via VVI-DETECT beforehand. In future work super-resolution techniques will be investigated to recover high-resolution images based on given low-resolution photos to allow the use of such material. Zhu et al.^90^ introduced an auspicious end-to-end CNN-based super-resolution network, entitled *Cross View Capture network* (CVCnet), outperforming state-of-the-art super-resolution methods. Furthermore, other data enhancement approaches, such as binary mask segmentation^55^ and/or contour detection^63,64^ of incoming images will be also of essential interest in the near future. Finally, the use of contextual knowledge is also a powerful and very promising avenue for improving FIN-PRINT, since killer whales have very distinctive and well documented social patterns and structures^15^. Such data can be used to actively adapt posterior probabilities, which in turn reduces the dimensionality of a potential classification hypothesis.

## Supplementary Information

Supplementary Information.

## Data Availability

Data to replicate the analyses are available from Bay Cetology and Fisheries and Oceans Canada upon reasonable request. Contact details can be obtained from the corresponding author. Upon acceptance, the code for FIN-PRINT will be made publicly available at https://github.com/ChristianBergler^91^, listing all single modules with a detailed description.
