# Supplementary material for: FIN-PRINT a fully-automated multi-stage deep-learning-based framework for the individual recognition of killer whales
Source: Sci Rep. 2021 Dec 6;11:23480. doi: 10.1038/s41598-021-02506-6 (PMC8648837; doi:10.1038/s41598-021-02506-6)
Supplement: Supplementary file 1 — Supplementary Information. [file 41598_2021_2506_MOESM1_ESM.pdf]

FIN-PRINT A Fully-Automated Multi-Stage Deep-Learning-Based Framework for the Individual Recognition of Killer Whales

Christian Bergler[1,*], Alexander Gebhard[1], Jared R. Towers[2,3], Leonid Butyrev[1], Gary J. Sutton[2,3], Tasli J. H. Shaw[2,3], Andreas Maier[1], and Elmar Nöth[1]

[1]Friedrich-Alexander-University Erlangen-Nuremberg, Department of Computer Science - Pattern Recognition Lab, Martensstr. 3, 91058 Erlangen, Germany
[2]Bay Cetology, 257 Fir street, Alert Bay, BC, V0N 1A0, Canada
[3]Pacific Biological Station, Fisheries and Oceans Canada, 3190 Hammond Bay Road, Nanaimo, BC, V9T 6N7, Canada
[*]christian.bergler@fau.de

Supplementary Information

Animal \ Year		2011		2012		2013		2014		2015		2016		2017		2018		∑		[%]	
single+multiple	single	s+m[1]	s[2]	s+m	s	s+m	s	s+m	s	s+m	s	s+m	s	s+m	s	s+m	s	s+m	s	s+m	s
**T019B**	**T019B**	349	229	167	112	249	196	441	338	318	191	249	187	341	209	229	143	**2,343**	**1,605**	1.398	1.849
**T060C**	**T123A**	162	110	207	74	249	236	151	164	174	107	248	264	639	273	182	206	**2,012**	**1,434**	1.201	1.652
**T109A**	**T012A**	386	250	331	226	263	272	318	199	305	402	53	56	271	0	76	0	**2,003**	**1,405**	1.195	1.619
**T123A**	**T060C**	131	134	86	146	299	196	193	112	149	112	390	178	418	394	297	125	**1,963**	**1,397**	1.171	1.610
**T065A**	**T011A**	111	27	28	178	194	126	289	70	267	58	182	247	342	372	325	174	**1,738**	**1,252**	1.037	1.443
**T036A**	**T049A1**	184	105	220	89	110	45	153	137	233	308	226	158	355	232	244	99	**1,725**	**1,173**	1.029	1.352
**T041A**	**T069C**	488	155	443	322	296	198	242	131	46	113	85	73	68	138	51	16	**1,719**	**1,146**	1.026	1.320
**T049A1**	**T102**	147	69	134	49	55	46	178	115	433	186	230	133	357	303	163	134	**1,697**	**1,035**	1.013	1.193
**T100C**	**T019C**	158	124	191	77	82	94	164	133	123	149	207	140	295	190	428	128	**1,648**	**1,035**	0.983	1.193
**T102**	**T041A**	96	251	63	333	61	154	145	125	342	17	243	20	479	43	211	22	**1,640**	**965**	0.979	1.112

[1] s+m = Number of images (#) including single and multiple labels (entire data pool) [2] s = Number of images (#) containing only a single label

Supplementary Table 1. The top-10 most commonly occurring individuals represented in images including single and multiple labels, as well as only single labels

Network \ Hyperparameter	Core Net-Arch[1]	Input-Size[2]	Pre-Train[3]	Loss[4]	Val-Met[5]	Val-Intv[6]	Lr[7]	Lr-Dec[8]	Net-Opt[9]	Es[10]	Bs[11]
**FIN-DETECT**	YOLOv3 and DarkNet53 Backbone	3× 416×416	ImageNet[86]	Mean Squared Error Binary Cross Entropy	F1-Score	1 Epoch	10^{-4}	1/2 after 4 Epochs	Adam ($\beta_1 = 0.9$, $\beta_2 = 0.999$)	7 Epochs	8 Samples
**VVI-DETECT**	ResNet34 with initial 9×9 Convolution	3× 512×512	—	Cross Entropy	Accuracy	2 Epochs	10^{-3}	1/2 after 8 Epochs	Adam ($\beta_1 = 0.5$, $\beta_2 = 0.999$)	24 Epochs	8 Samples
**FIN-IDENTIFY**	ResNet34 with initial 9×9 Convolution	3× 512×512	—	Cross Entropy	Accuracy	2 Epochs	10^{-3}	1/2 after 8 Epochs	Adam ($\beta_1 = 0.5$, $\beta_2 = 0.999$)	20 Epochs	8 Samples

[1] Core Net-Arch = Core Network Architecture – The core structure on which the respective model is based
[2] Input-Size = Network Model Input Size – Pre-processed RGB-sub-images building the network input
[3] Pre-Train = Pre-Trained Network Model – Initialization with pre-trained weights
[4] Loss = Loss Function – Loss criterion used for training the network
[5] Val-Met = Validation Metric – Validation criterion to evaluate model performance
[6] Val-Intv = Validation Interval – Number of epochs after which the model is evaluated on the validation set.
[7] Lr = Learning Rate – Initial learning rate utilized for network training
[8] Lr-Dec = Learning Rate Decay – Decay factor and number of epochs without any improvements on the corresponding validation metric
[9] Net-Opt = Network Optimization Algorithm – Model optimizer and corresponding β values
[10] Es = Early Stopping Criterion – Number of epochs without any improvements on the corresponding validation metric, after which the training is stopped
[11] Bs = Batch Size – Number of samples within a single batch

Supplementary Table 2. Overview about all relevant network hyperparameters utilized for training FIN-DETECT, VVI-DETECT, and FIN-IDENTIFY

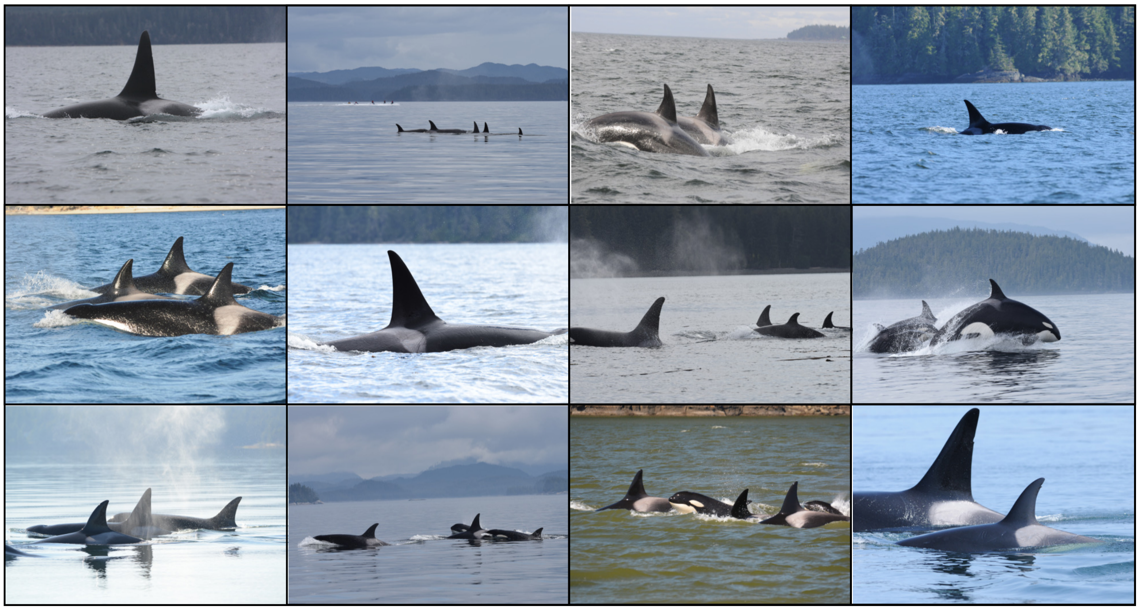

Supplementary Figure 1. Examples images of Bigg's killer whales from the original dataset (2011-2018)

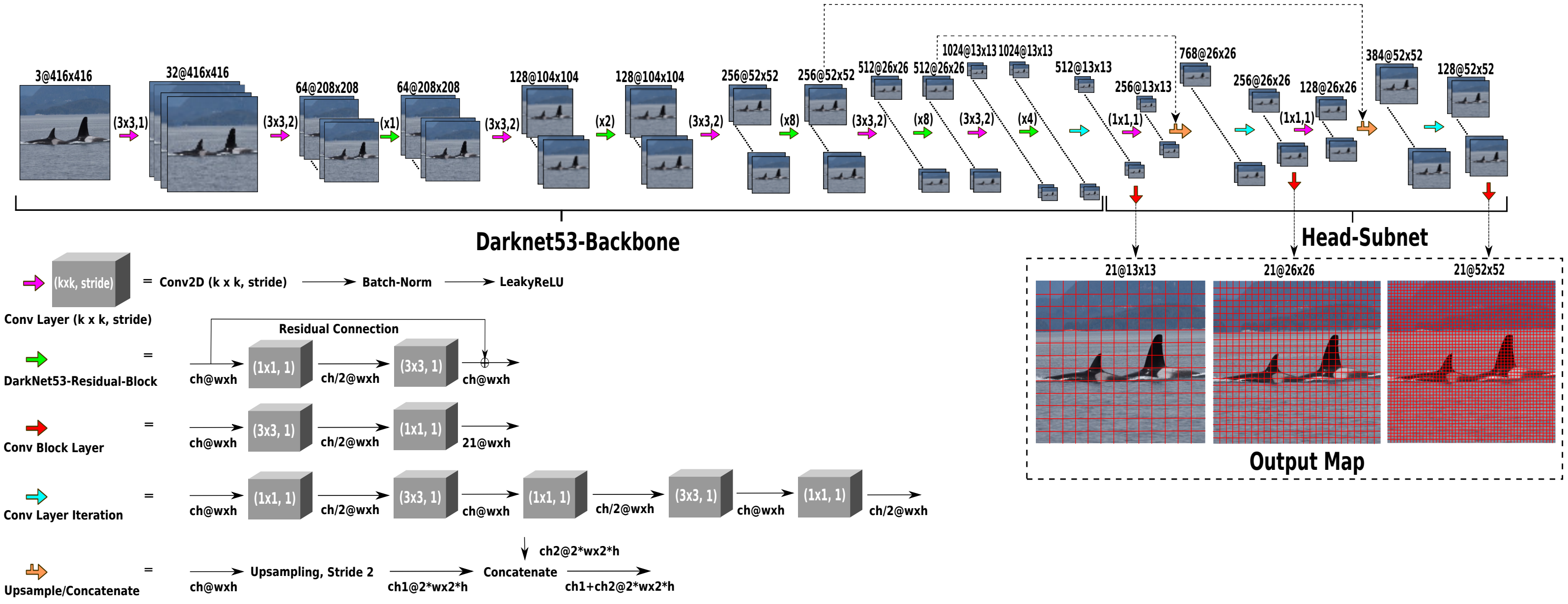

Supplementary Figure 2. FIN-DETECT, a dorsal fin/saddle patch detection model, utilizing the original YOLOv3[76,77] object detection network architecture, extended by additional functionalities (illustration recreated after Zhang et al.[83])

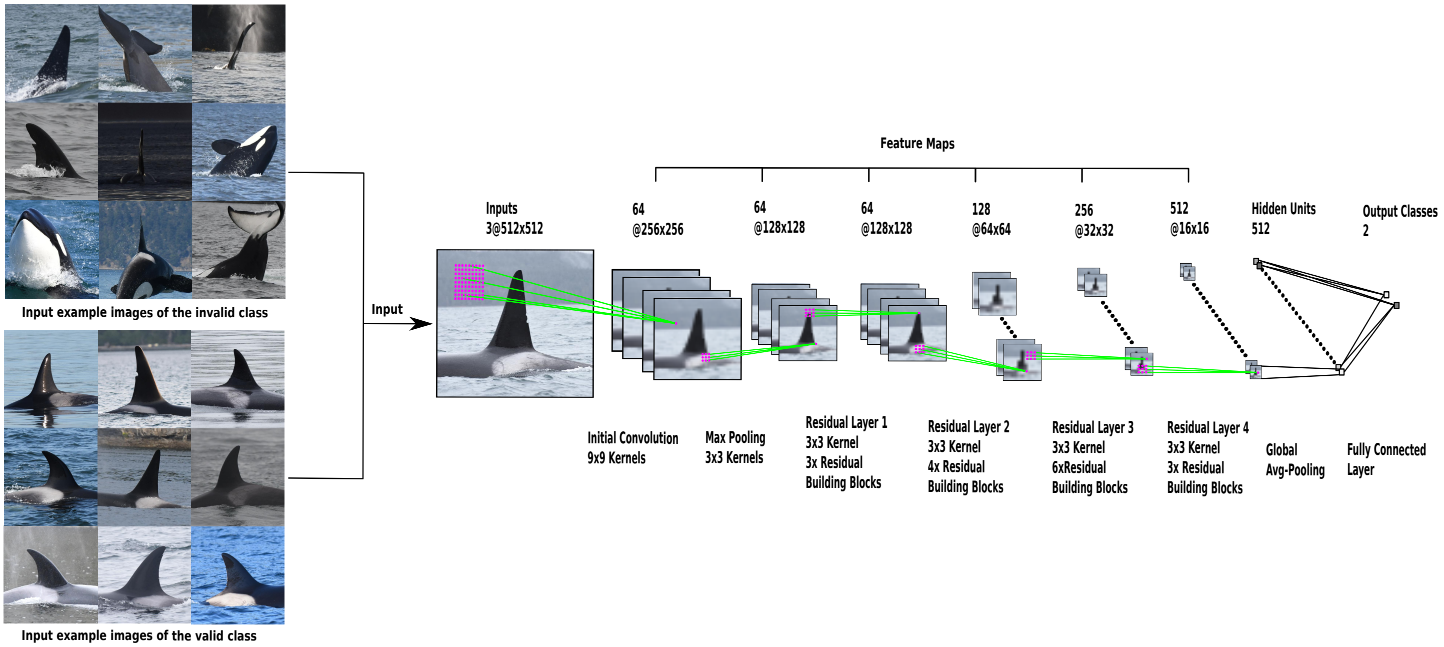

Supplementary Figure 3. VVI-DETECT, a ResNet34[78]-based binary classification CNN, distinguishing between valid versus invalid (VVI) identification images

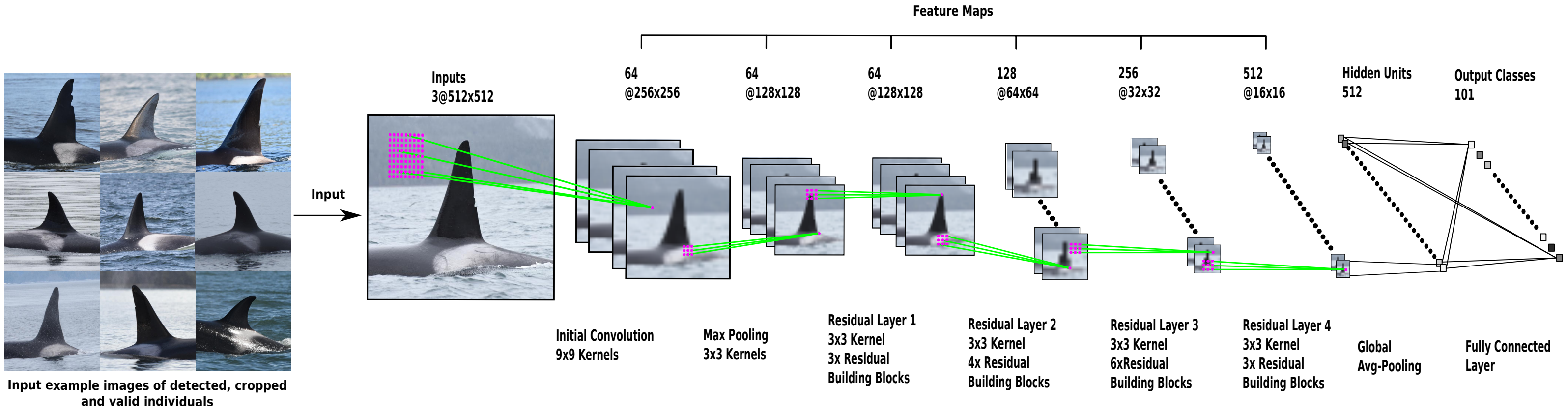

Supplementary Figure 4. FIN-IDENTIFY, describes a ResNet34[78]-based 101-class individual classification network.
